# A non-rigid registration method for the analysis of local deformations in the wood cell wall

**DOI:** 10.1186/s40679-018-0050-0

**Published:** 2018-01-22

**Authors:** Alessandra Patera, Stephan Carl, Marco Stampanoni, Dominique Derome, Jan Carmeliet

**Affiliations:** 1 Swiss Light Source, Paul Scherrer Institute, Villigen, Switzerland; 2 Centre d’Imagerie BioMedicale, Ecole Polytechnique Federale de Lausanne, 1015 Lausanne, Switzerland; 3 EMPA, Swiss Federal Laboratories for Materials Science and Technology, Laboratory for Multiscale Studies in Building Physics, Überlandstrasse 129, 8600 Dübendorf, Switzerland; 4 ETH Zurich, Chair of Building Physics, Stefano-Franscini-Platz 1, Zürich Hönggerberg, 8093 Zurich, Switzerland; 5 ETH Zurich, Institute for Biomedical Engineering, Gloriastrasse 35, 8092 Zurich, Switzerland

**Keywords:** Image registration, B-spline function, Free form deformation, Spruce wood, X-ray tomography, Equivalent strain

## Abstract

This paper concerns the problem of wood cellular structure image registration. Given the large variability of wood geometry and the important changes in the cellular organization due to moisture sorption, an affine-based image registration technique is not exhaustive to describe the overall hygro-mechanical behaviour of wood at micrometre scales. Additionally, free tools currently available for non-rigid image registration are not suitable for quantifying the structural deformations of complex hierarchical materials such as wood, leading to errors due to misalignment. In this paper, we adapt an existing non-rigid registration model based on B-spline functions to our case study. The so-modified algorithm combines the concept of feature recognition within specific regions locally distributed in the material with an optimization problem. Results show that the method is able to quantify local deformations induced by moisture changes in tomographic images of wood cell wall with high accuracy. The local deformations provide new important insights in characterizing the swelling behaviour of wood at the cell wall level.

## Background

**Fig. 1:**
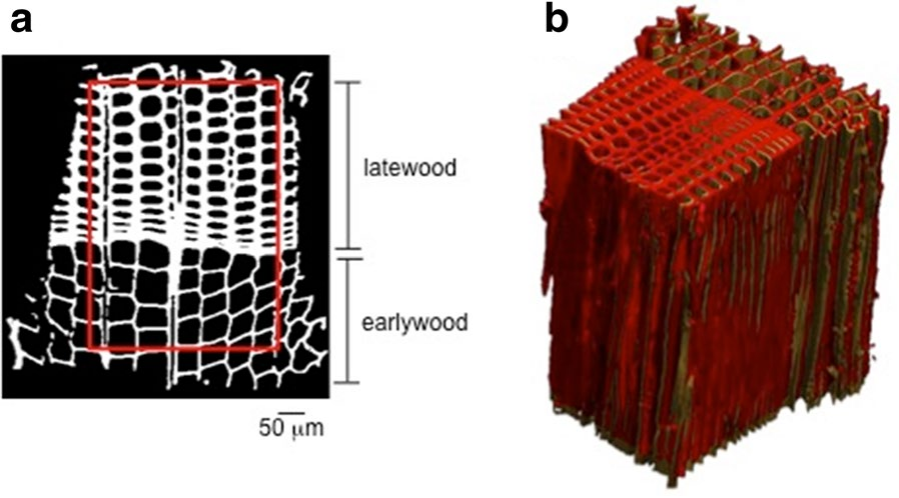
**a** Cross-sectional view of spruce wood sample consisting in two tissues with different porosity, i.e. latewood and earlywood. The ROI used for analysis is shown with the red box. **b** 3D comparison of the volumes at the two RH states: 25%RH in yellow and 85%RH in red

Wood, a cellular biological material, swells or shrinks due to the adsorption or desorption of water in the hygroscopic domain. While sorption occurs within the cell wall material, the cellular organization of wood leads to significant deformations in the transversal directions of wood, i.e. tangentially and radially to the growth rings, with very little swelling in the longitudinal direction. Growth rings are made of low density spring wood and denser summer wood, yielding cell lumen and wall dimensions to vary over more than one order of magnitude. Most cells, the tracheids, are vertical in the trunk but 5–10% of the cells, named rays, are positioned horizontally linking radially the cells across growth rings. Given this geometrical variability, an analysis of wood deformations at the cellular scale could elucidate whether swelling is regular or is accompanied by local deformations. Tridimensional characterization of swelling strains in wood tissues is rarely documented. X-ray tomography, a non-invasive method, is retained to image tridimensionally the cellular structure, at different states of swelling or shrinkage induced by relative humidity changes, an example of wood structure with earlywood and latewood, scanned at two relative humidity (RH) values, i.e. 25% RH and 85% RH, is shown in Fig. 1. In Derome et al. [5] and in Patera et al. [25], the swelling/shrinkage of wood has been documented in 3D by adopting an affine registration model as good approximation for describing the deformations of wood cell wall due to moisture changes. However, this model fails to identify the local deformations, thus resulting not exhaustive in the characterization of the hygro-mechanical behavior of wood. This paper presents a quantitative approach to precisely capture the local deformations of wood cellular structure in high-resolution tomographic datasets. The proposed method represents a modification of an existing non-rigid image registration algorithm to be adapted to the specific case study. However, the same method can be applicable to a variety of materials that present a complex structure such as wood. Before describing our approach for resolving quantitatively the local deformation in wood cell wall, a general overview of the documented image registration algorithms is briefly given in the following paragraph. Furthermore, the B-spline based non-rigid registration technique is detailed described prior defining the suggested improvements to make it suitable for material science applications. The method is then validated by means of finite element modelling comparison and applied on images of wood cell wall acquired at two relative humidity states.

## Image registration problem: a general overview

Image registration is a method to map two different images, which are acquired with the same or different experimental setups. Due to the importance of image registration in various application areas and given its complicated nature, a large number of image registration algorithms have been developed in the past. An exhaustive review of general-purpose image registration methods can be found in Brown [2] and in Wyawahare et al. [33]. Applications of image registration in the medical field include combining data from different modalities e.g., computer tomography (CT) and magnetic resonance imaging (MRI), to obtain more complete information about the patient, monitoring tumour growth [33], treatment verification [11, 12, 30], computer-aided diagnosis and disease following-up [15]; surgery simulation [22]; atlas building and comparison [13]; radiation therapy [8, 17]; anatomy segmentation [4, 7, 9, 10, 16, 20, 34] and image subtraction for contrast-enhanced images [19]. In contrast, much less algorithms for image registration are nowadays available for material applications. To allow introducing the basic idea of the new algorithm, aimed at capturing the local deformations of cellular materials, such as wood, a general mathematical description of image registration method is first given.

In image registration, the deformed image, also called *moving image I*_*M*_(*x*), is transformed to be aligned to the reference or original image, called *fixed image I*_*F*_(*x*). Both images have dimensions *s* and are defined in their spatial domain: $\varOmega_{F} \subset {\mathbb{R}}^{d}$ and $\varOmega_{M} \subset {\mathbb{R}}^{d}$ for fixed and moving images, respectively. In general, the transformation is defined as a mapping from the moving to the fixed image, i.e. $T:\varOmega_{F} \subset {\mathbb{R}}^{d} \to \varOmega_{M} \subset {\mathbb{R}}^{d}$. The transformation that matches *I*_*M*_(*x*) to *I*_*F*_(*x*) is defined as:

1 $$T\left( x \right) = x + U(x)$$

where *U*(*x*) is the displacement that makes $I_{M} (x + U(\varvec{x}))$ to be aligned to *I*_*F*_(*x*). The goodness of alignment is evaluated by a distance or similarity measure ${ \mathcal{S}}$, such as the correlation ratio, the sum of squared differences (SSD), or the mutual information (MI).

Commonly, registration is a problem of optimization in which a cost function ${\mathcal{C}}$ has to be minimized, resulting in $\hat{T}$:

2 $$\hat{T} = { \arg }\,\mathop {\hbox{min} }\limits_{T} {\mathcal{C}}(T;I_{F} ,I_{M} )$$

where $\hat{T}$ represents the set of points of the transformation between fixed and moving images for which the given cost function attains its minimum value.

The problem of the identification of the non-rigid transformation $T$ becomes easily ill-posed and, therefore, a regularisation or penalty term ${\mathcal{P}}$ constraining the transformation *T* is introduced. Then the cost function is as follows:

3 $$C\left( {T;I_{F} ,I_{M} } \right) = - {\mathcal{S}}\left( {T;I_{F} ,I_{M} } \right) + \gamma {\mathcal{P}}\left( T \right)$$

where *γ* weights similarity against regularity. The cost function is described by the similarity term when *γ* tends to zero. A similarity measure is a function that takes two input images as parameters and computes a numerical value that quantifies the extent to which the two images are similar. The regularity term ${\mathcal{P}}\left( T \right)$ is designed to penalize control points displacements that potentially lead to naturally implausible deformations.

Transformations used to align two images can be global or local. A global transformation is given by a single equation, which maps the entire image. One global method is the affine registration model, which allows to quantify the affine strains along the three orthotropic directions of wood. However, this model fails to identify the local deformations [5, 25]. In this paper, an approach to detect and quantify local deformations in the cellular tissues using a non-rigid registration model is proposed. Any plane through wood cellular structure shows a form, which can be represented by a free-form surface with the aim of tracking its deformation during free swelling. This freeform surface can be determined using control points connected together by a mesh. The surface is approximated using a control mesh guaranteeing a certain level of smoothness. Many representations of free-form surface exist in the literature [29] and an approach of representing free-form deformations based on B-splines is used [28]. The approach proposed in this work has been adapted to the specific case study of wood cellular material and implemented in Matlab. It is based on an existing method implemented by Rueckert, as described in the following.

### B-splines based non-rigid registration method: an elegant formulation

**Fig. 2:**
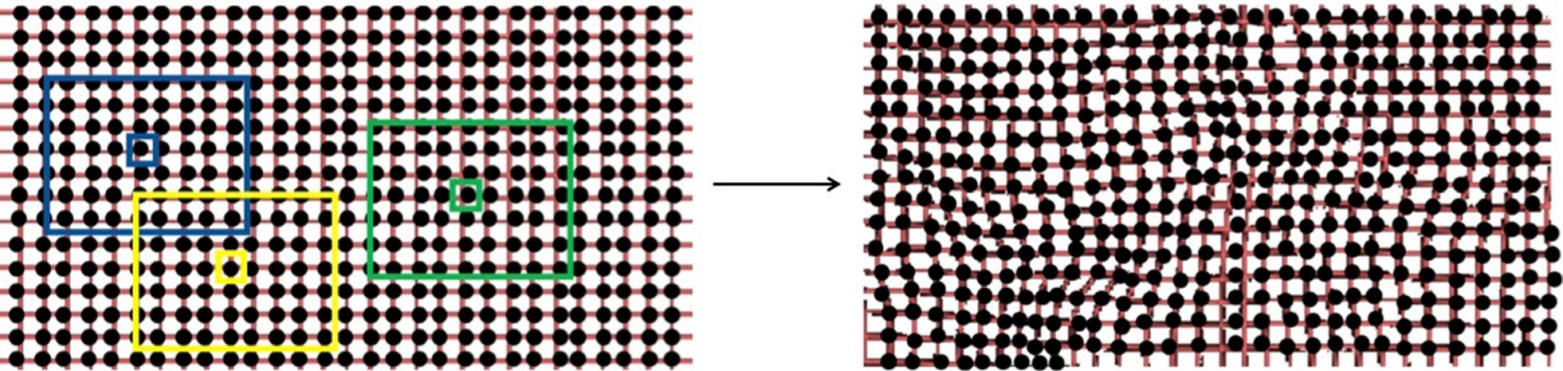
Example of deformable mesh in which each point is locally controlled, as shown, for example, in the blue, yellow and green boxes

The local transformation *T*_local_(*x*, *y*, *z*) captures the local deformations in the object. An elegant way to describe local deformations avoiding difficult parameterization methods has been introduced by Rueckert et al. [28]. The model is based on B-splines [31] and it is known as the Free Form Deformation (FFD) model. FFD is a technique for manipulating any shape in a free-form manner, as shown in Fig. 2. Basically, an object is deformed by manipulating an underlying mesh of control points. The local transformation is described in the volume domain $\varOmega = \left\{ {(x,y,z)|0 \le x < X, 0 \le y < Y,0 \le z < Z} \right\}$ as a mesh of control points Π_*i*,*j*,*k*_ with uniform spacing *δ* and with number of elements *N* = *n*_*x*_ × *n*_*y*_ × *n*_*z*_. The transformation function *T*_local_(*x*, *y*, *z*) is written as the 3-D tensor product of 1-D cubic B-splines and is defined in terms of the control points Π_*i*,*j*,*k*_:

4 $$T_{\text{local}} \left( {x,y,z} \right) = \sum\limits_{l = 0}^{3} {\sum\limits_{m = 0}^{3} {\sum\limits_{n = 0}^{3} {B_{l} (u)B_{m} (v)B_{n} (w)\varPi_{i + l,j + m,k + n} } } }$$

where $i = \left\lfloor {x/n_{x} } \right\rfloor - 1,\,j = \left\lfloor {y/n_{y} } \right\rfloor - 1,\,k = \left\lfloor {z/n_{z} } \right\rfloor - 1$ and where ⌊⌋ means the floor of the number (i.e. the largest integer less than or equal to the number). The variables *u*, *v*, *w* are defined as: $u = x/n_{x} - \left\lfloor {x/n_{x} } \right\rfloor ,\,v = y/n_{y} - \left\lfloor {y/n_{y} } \right\rfloor w = z/n_{z} - \left\lfloor {z/n_{z} } \right\rfloor .$ $B_{1}$ represents the *l*th basis function of the B-spline, as defined below:

5 $$\begin{aligned} B_{0} \left( u \right) &= \left( {1 - u} \right)^{3} /6 \\ B_{1} \left( u \right) &= (3u^{3} - 6u^{2} + 4)/6 \\ B_{2} \left( u \right) &= ( - 3u^{3} + 3u^{2} + 3u + 1)/6 \\ B_{3} \left( u \right) &= u^{3} /6 \\ \end{aligned}$$

The control points Π_*i*,*j*,*k*_ are the unknown parameters of the B-spline FFD. The level of the non-rigid transformation depends on the resolution of the mesh of the control points. The spacing between the control points determines the resolution of non-rigid registration, i.e. a large spacing or low resolution results in a more global estimation of the deformations, compared to a smaller spacing (higher resolution) which models highly local deformations. At the same time, the number of control points determines the number of degrees of freedom and the computational complexity. The B-spline grid is constructed with the method of Lee et al. [18].

The total transformation in the object is defined as the sum of the global and local transformation.

6 $$T\left( {x,y,z} \right) = T_{\text{global}} \left( {x,y,z} \right) + T_{\text{local}} \left( {x,y,z} \right)$$

The parameters of the global and local transformations are determined by solving the optimization problem, i.e. by minimising the cost function defined in Eq. 3. A penalty term is introduced in Eq. 3 to constrain the local transformation and ensure smoothness. This term is described by Wahba [32] as follows:

7 $${\mathcal{P}}\left( T \right) = \frac{1}{V}\int_{0}^{X} {\int_{0}^{Y} {\int_{0}^{Z} {\left[ {\left( {\frac{{\partial^{2} T}}{{\partial x^{2} }}} \right)^{2} + \left( {\frac{{\partial^{2} T}}{{\partial y^{2} }}} \right)^{2} + \left( {\frac{{\partial^{2} T}}{{\partial z^{2} }}} \right)^{2} + 2\left( {\frac{{\partial^{2} T}}{\partial xy}} \right)^{2} + 2\left( {\frac{{\partial^{2} T}}{\partial xz}} \right)^{2} + 2\left( {\frac{{\partial^{2} T}}{\partial yz}} \right)^{2} } \right]} } } {\text{d}}x\,{\text{d}}y\,{\text{d}}z$$

with *V* denoting the volume of the image domain. The penalty term of a cost function is equal to zero in the case of an affine transformation. In Eq. 3, the similarity term is evaluated by comparing the histogram, hist, of *I*_*F*_ and *I*_*M*_. As a general rule, mutual information (e.g., the degree of dependence of one image with respect to the other one, Pluim et al. [27]) is applied if

8 $$\mathop \sum \nolimits \left| {{\text{hist}}\left( {I_{M} } \right) - {\text{hist}}\left( {I_{F} } \right)} \right| > 0.25$$

In the specific case study of this work, the difference between the two histograms of grey levels of the reference and moving images is smaller than 0.25 and the goodness of alignment cannot, therefore, be evaluated with the mutual information. Thus, the similarity measure is evaluated with the SSD.

## Methods

### A modified version of the B-splines based non-rigid registration algorithm for material science applications: the wood case study

In general, the deformation of wood contains a non-rigid component so that affine transformations alone are not sufficient to describe local deformations in wood tissues, subjected to free swelling due to water vapour adsorption, as identified, but not quantified yet, in Derome et al. [5] and Patera et al. [25]. Therefore, the transformation analysis includes both the affine and non-rigid components, as shown in Eq. 6. The global transformation *T*_global_(*x*, *y*, *z*) is the affine transformation resulting from the affine registration model, as described in Derome et al. [5] and Patera et al. [25].

The algorithm, proposed by Rueckert et al. [28] and described above, is modified to improve the performance of the FFD for describing local deformations in complex cellular materials, such as wood. Most of the algorithms presented in literature, such as the one applied in this work, are based on the histogram of grey levels. The basic and simple idea of this modified version is to introduce some morphological operations in the original method to *guide* the algorithm in recognising typical features in complex structures, such as wood.

**Fig. 3:**
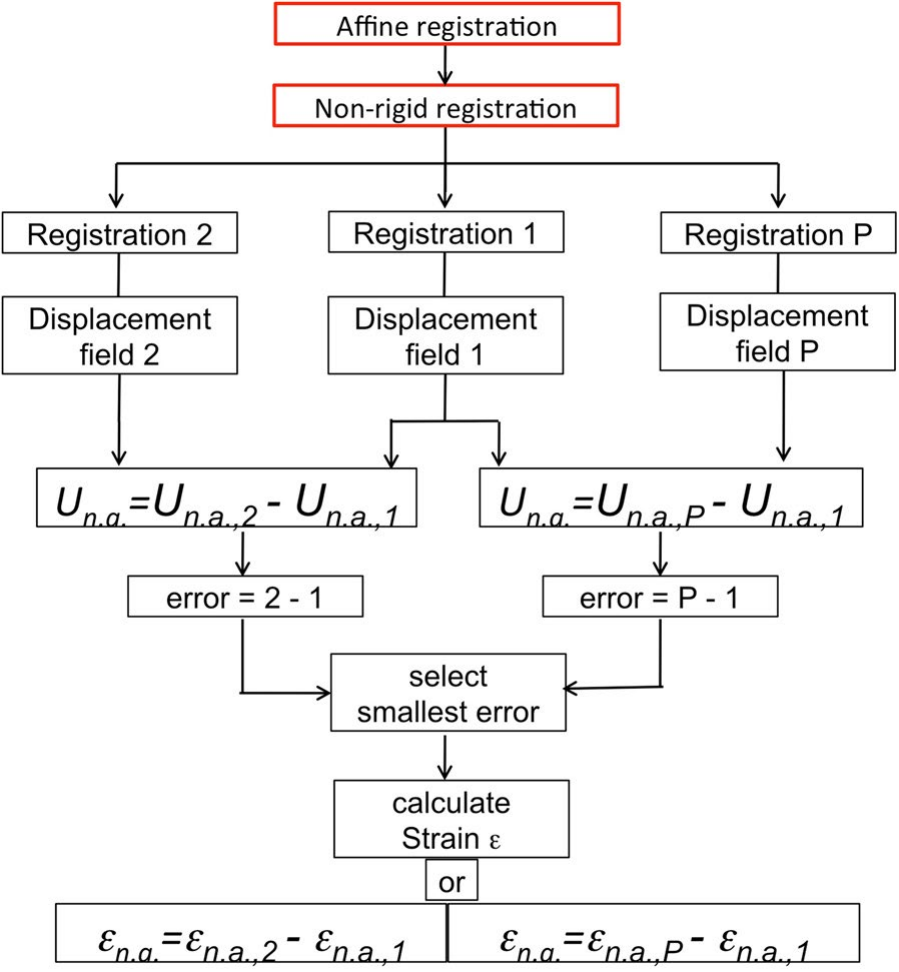
Overview of the non-rigid registration algorithm with the three methods described in the text

As an overview of the non-rigid registration method, a flowchart is given in Fig. 3. Three approaches for non-rigid registration are used to register the *moving image I*_*M*_(*x*) and the *fixed image I*_*F*_(*x*). The two first registration approaches perform registration directly on the images, i.e., on the intensity of the grey values. We refer to these two approaches as ‘Registration 1’ and ‘Registration 2’. ‘Registration 1’ is a rough and fast estimation of non-rigid deformations performed on smaller size images, where the B-spline functions are largely constrained (increasing the weight *γ* of the penalty term in Eq. 3) to describe the more global deformations in the material. ‘Registration 2’ is performed directly on the original images with increasing the freedom of the B-spline grid, i.e. decreasing the weight coefficient *γ* of the penalty. In this way, the local misalignment can be more easily detected, although more artefacts coming from the high freedom of the B-spline grid can arise. To prevent these artefacts problems, the local deformations are defined by subtracting ‘Registration 2’ from ‘Registration 1’ and the local transformation is calculated. However, there are cases in which these methods do not give the optimal solution. For this reason, a third registration technique is introduced. The third approach for non-rigid registration is called point-based registration or ‘Registration P’. In this case, the input consists in a set of points in the two images. Different techniques for the detection of control point pairs in the images are used, which are named Manual, Map, Skeleton, Harris or Edges techniques. The manual selection technique consists in selecting the initial pairs in the two images manually. All the other techniques are automatic. Map is a simple procedure of tracking the borders of features using binary images and giving the coordinates of the borders as output. Skeleton follows a skeletonization procedure consisting in the extraction of a region-based shape feature which represents the general structure of an object. Harris is a method for calculating and displaying the feature points as Harris corners [14] and, finally, edges is based on the Canny edges detection method [3]. One of these methods is initially used to extract feature points in both the fixed and moving images. The registration algorithm is performed on pair of control points instead of the image histograms. After detection, it is important to check that corresponding feature coordinates are found in both images. A normalized cross-correlation function is thus introduced to adjust each pair of control points. The algorithm moves the position of a control point by up to four pixels, to adjust the coordinates with an accuracy up to one-tenth of a pixel.

Then, the initial image pairs $\left\{ {\varPi^{i} \left( {I_{F} } \right), \varPi^{k} \left( {I_{M} } \right)} \right\}_{i,k = 1 \ldots n}$ are added, in both images, to the points of the artificial grids $\left\{ {\varPi^{j} \left( {G_{F} } \right), \varPi^{l} \left( {G_{M} } \right)} \right\}_{j,l = 1 \ldots m}$ to obtain Π_*F*_ and Π_*M*_, as:

9 $$\left\{ {\begin{array}{l} {\Pi _{F} = \left\{ {\Pi ^{i} \left( {I_{F} } \right),\Pi ^{j} \left( {G_{F} } \right)} \right\}_{\begin{subarray}{l} i = 1 \ldots n \\ j = 1 \ldots m \end{subarray} } } \\ {\Pi _{F} = \left\{ {\Pi ^{k} \left( {I_{M} } \right),\Pi ^{l} \left( {G_{M} } \right)} \right\}_{\begin{subarray}{l} k = 1 \ldots n \\ l = 1 \ldots m \end{subarray} } } \\ \end{array} } \right.$$

**Fig. 4:**
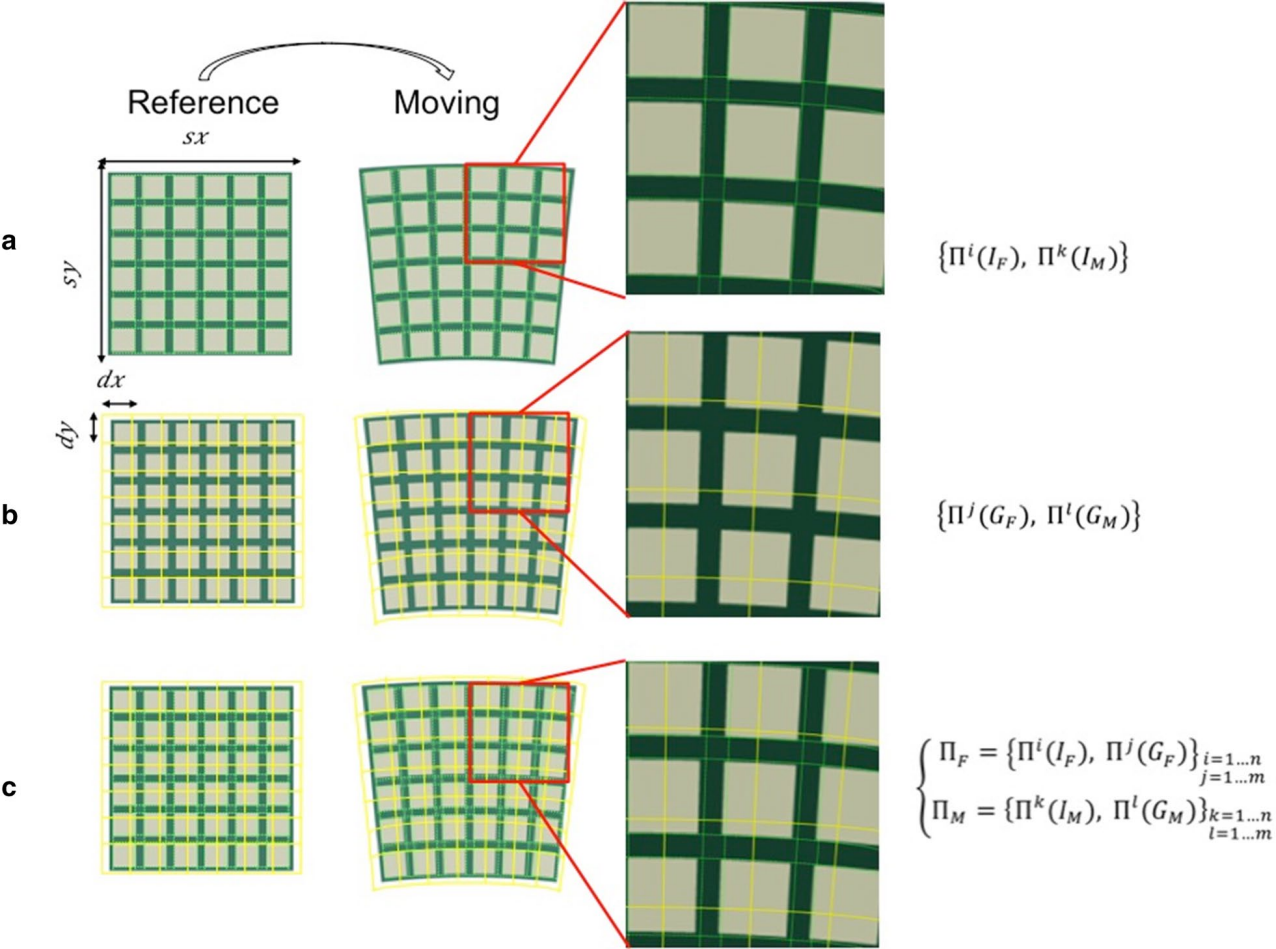
Graphical illustration of the different steps representing the two key-features of non-rigid registration algorithm based on points. On the right, a zoom on a squared region of interest is presented to visualise the described procedure. **a** The feature points (or coordinates) are extracted on the reference and moving images. **b** An artificial grid is added on both images and, finally, **c** the features points are summed to the nodes of the artificial grid in both, reference and moving images

A polynomial or affine transformation is used to transform the artificial grid matching Π^*j*^(*G*_*F*_) with Π^*l*^(*G*_*M*_). The initial pairs or feature points in the two images are selected in such a way to ensure a matching between image features using correlation. A graphical illustration of the point registration method, illustrating how the feature points are added to the artificial grid coordinates, is given in Fig. 4.

One of the major drawbacks of such non-rigid registration method is related to the high-degree of freedom-inducing artefacts which is given to the B-spline functions to capture all potential local deformations in the structure. A way to prevent such artefacts is to include more constraints on the transformation. However, this becomes at finer grid resolutions. To overcome these difficulties, the solution proposed in this work is to make a comparison between two registration types and to consider, as final result, the image difference between ‘Registration 1’, considered as the reference since it includes more constraints, and ‘Registration 2’ or ‘Registration P’, depending on the case study.

The error is defined as the pixel difference between the two images after non-rigid registration. The pair of registration images with the smallest difference is considered for the final calculation of the local deformations and non-rigid strains. This step ensures the selection of the best non-rigid registration method for each specific region of interest studied in the volume. Once the optimization problem is solved by minimization of the cost function, the displacement field is determined and the local strain fields are evaluated and plotted.

The algorithm runs for a stack of images in a loop, performing a 2D registration image by image. In the case study of swelling deformations in softwood tissues, a 2D registration is sufficient to define the local deformations as wood swelling is predominantly occurring along tangential and radial directions. For each registration step (i.e. ‘Registration 1’, ‘Registration 2’ and ‘Registration P’), the non-rigid (briefly, n.a. to differentiate from the affine *a.*) strain fields are calculated from the gradient of the displacement field (*U*_*x*_, *U*_*y*_) in each direction (${{\varepsilon_{\text{n.a.}}}_{x}}, {{\varepsilon_{\text{n.a.}}}_{xy}}, {{\varepsilon_{\text{n.a.}}}_{y}}$) and the equivalent strain is calculated using a simplification of the von Mises relationship (von Mises [23]), valid in this case study:

10 $$\varepsilon_{\text{n.a.}} = \sqrt {{{\varepsilon_{\text{n.a.}}}_{x}^{2}} + 2 \times {{\varepsilon_{\text{n.a.}}}_{xy}^{2}} + {{\varepsilon_{\text{n.a.}}}_{y}^{2}}}$$

The equivalent strain is used to describe the deformation intensity in wood. More details on strain tensors calculation can be found in Abd-Elmoniem et al. [1].

As previously described, the non-rigid registration problem can be then solved by performing first the intensity-based methods (‘Registration 1’ and ‘Registration 2’) and then the point-based method (‘Registration P’). The two intensity-based registrations methods are the critical steps of the algorithm as they incorporate the initial optimisation and minimisation problem in a sequential loop.

Rueckert et al. [28] describe the optimization problem in terms of minimizing a cost function, as given in Eq. 3, where the optimization proceeds in several steps to improve the computational efficiency. First, the affine transformation *T*_global_(*x*,*y*,*z*) is optimized, which corresponds to optimizing the similarity between the two images, where the penalty term of the cost function in (7) is zero. During the subsequent stage, the non-rigid registration parameters are optimized. In each stage, a simple iterative steepest descent technique is used stepping in the direction of the gradient vector with a certain step size. The algorithm stops when a local minimum of the cost function is found, given by the condition that $\left\| {\nabla {\mathcal{C}}} \right\| \le \chi$ for some small positive value of *χ*. The minimization loop based on the steepest descent technique is implemented within a line search strategy. As line search strategy, two methods are used: the first is a simple one based on a parametric function; the second is a normal line search method with Wolfe conditions. A detailed description of line search strategy can be found in Nocedal and Wright [24].

As mentioned above, the algorithm is implemented in 2D as the deformations in wood occur almost only along the tangential and radial directions. Therefore, before applying the non-rigid registration algorithm, a set of slices at the same plane of fixed and moving images are selected. Then, the optimal parameters for the three registrations methods, ‘Registration 1’, ‘Registration 2’ and ‘Registration P’, are determined. Finally, the algorithm runs in a loop over the whole stack of slices.

## Validation of the algorithm by comparison with a finite element model

**Fig. 5:**
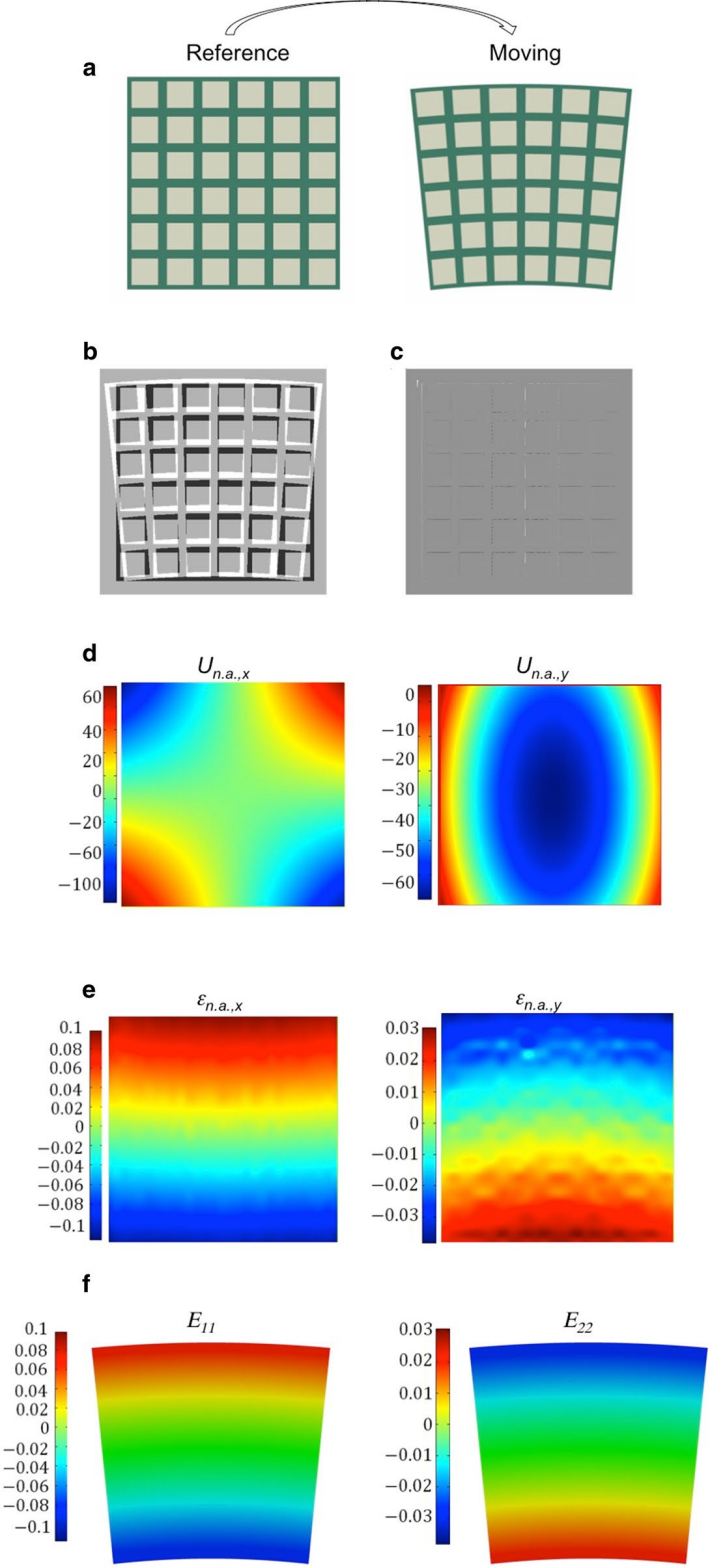
Example of application of the algorithm on an artificial homogeneous image (**a**) where the grid is imposed to highlight the deformations and mismatches. The difference between reference and moving images is shown before registration (**b**), then after non-rigid registration (**c**). In **d** the non-rigid displacement fields in the two directions and in **e** the non-rigid strains in the *x*–*y* plane are mapped on the reference image. **f** Strain fields plotted on the deformed image as result of finite element simulation, under the assumption of homogeneous material. The strain in *x*-direction (E11) is on the left side, the strain calculated in *y*-direction (E22) is on right side

The performance of the algorithm is validated in 2D using a pair of images, e.g. a fixed image I_F_ and a moving image I_M_, generated specifically for this validation exercise. A homogeneous sample of square configuration undergoes bending, which is a pure non-rigid deformation, and the deformations are calculated by finite element method, as shown in Fig. 5a where a grid is drawn for visualisation of the deformation. In this example, the steepest descend method is selected to solve the optimization problem, since a better solution is found with this method compared to line search methods of optimization. Additionally, *γ* = 0.01 in ‘Registration 1’ and *γ* = 0.001 in ‘Registration 2’. In ‘Registration P’, ‘Map’ is chosen as the method for point extraction. When the algorithm finds a minimum, the refinement loop is ended. The error between the reference and the deformed images is calculated for each registration type. In this case, the error is expressed in terms of number of pixels, normalised over the total amount of pixels in the region. The error obtained for ‘Registration P’, 0.006, is smaller than the error calculated with ‘Registration 2’, 0.009. Therefore, ‘Registration P’ is chosen for the calculation of the deformation fields and of the local strains shown in Fig. 5d, e.

The local strains calculated with the modified algorithm can be compared with the results of the simulation with finite element (Fig. 5f) obtained with the software Abaqus FEA. A good agreement between the results of simulation and registration algorithm is observed. The strains show in the same range of values and distribution over the surface. Only is the non-rigid strains, in the *y*-direction, we see some fluctuations.

## Results and discussion

The non-rigid registration algorithm presented in this paper is used for documenting the occurrence of local deformations during swelling of the complex cellular structure of softwood. The focus is to investigate the moisture-induced deformations in tissues from spruce wood, namely *Picea abies* (L. Karst). The algorithm is applied on tomographic datasets of wood with a voxel pitch equal to 0.8 μm, acquired at the Centre for X-Ray Tomography of the Ghent University (UGCT) in Belgium [6, 21]. The analysis is performed on a wood sample of cross-section dimensions of approximately 500 × 700 μm^2^ which presents a combination of tissues, named earlywood and latewood, with different porosities (≈ 78% for earlywood and 45% for latewood) and hygro-mechanical behaviour (anisotropic swelling in earlywood and more isotropic swelling in latewood). The sample is scanned at two relative humidity (RH) values, referred as dry state, i.e. 25% RH, and wet state, i.e. 85% RH, as shown in Fig. 1b. Wood swells when exposed to an increase in RH and the typical deformations observed in the cellular structure of wood samples has been described globally. From previous [5, 25] and recent work [26], it is known that these X-ray measurements are reproducible as no deformation is seen between the CT datasets acquired at start and end of the sorption–desorption sequences. The aim of this study is to quantify these deformations also locally using non-rigid registration.

**Fig. 6:**
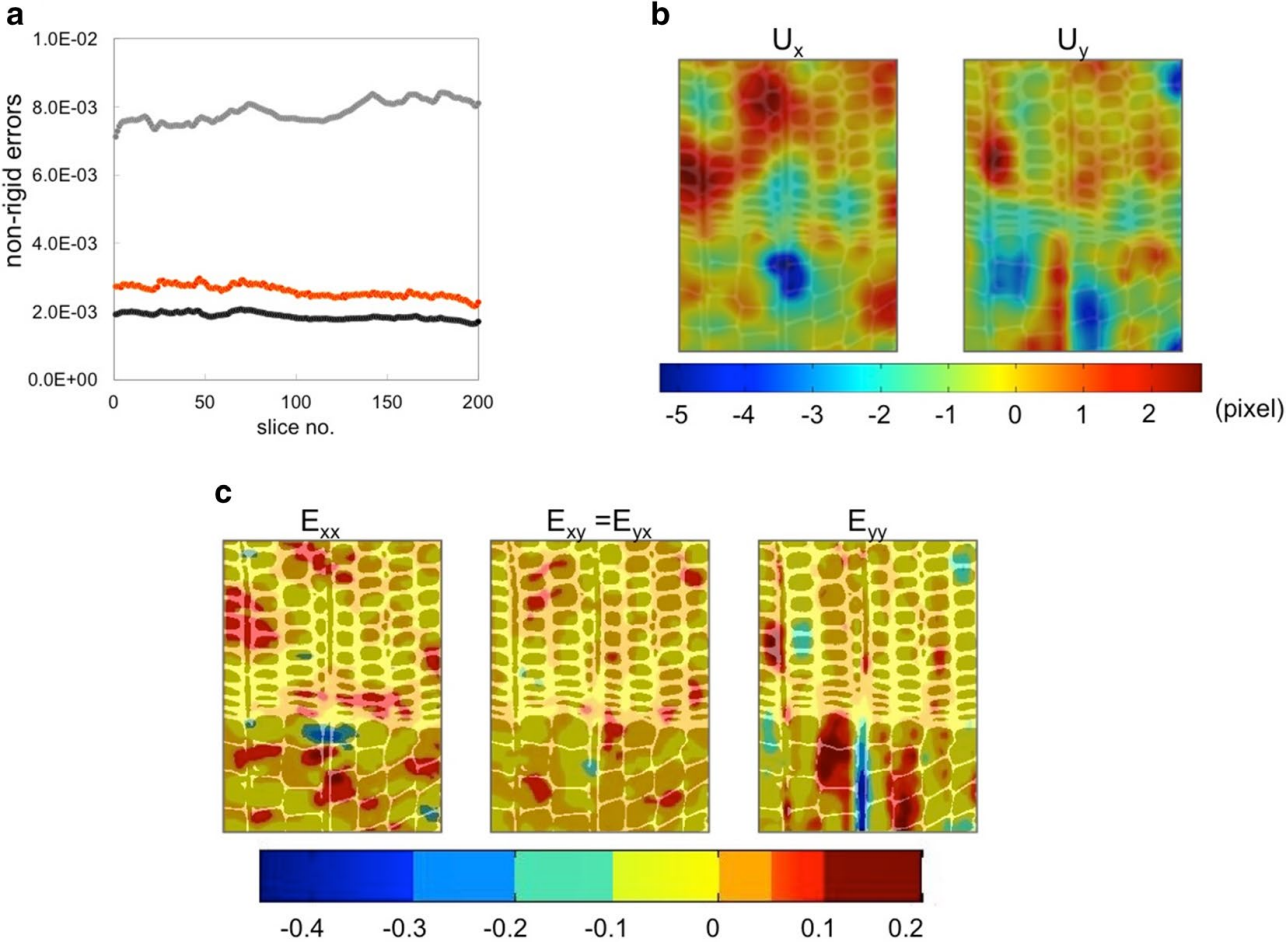
**a** Errors between reference and registered images after non-rigid registration for each slices of the volume, using an intensity-based method for R_1_ (grey) and R_2_ (black) estimation and a point-based registration R_P_ (orange). **b** Displacement fields in one slice of the wood sample in pixels (1 pixel = 0.8 μm), **c** 2D map of total strains (affine plus non-rigid) on the reference images

From the 3D datasets, 200 cross-sectional slices are selected and further cropped to a region of interest (ROI) of the cellular structure, as shown in Fig. 1a. Two datasets are thus obtained, using the images acquired at 25% RH as the fixed images and the ones at 75% RH at the moving images. After the affine registration, the non-rigid registration proceeds as follows. The registration approach based on the grey values intensity with the steepest descend method allows to compare the performance of the B-spline algorithm in two cases: the first on the resized volumes for a fast estimation (‘Registration 1’), where the B-spline functions are constrained to less freedom using a value of *γ* = 1.0*E*−2, while the second case, ‘Registration 2’, consists in giving more freedom to the functions with a *γ* = 1.0*E*−4. Additionally, the point-based registration algorithm (‘Registration P’) is applied with the method named ‘Map’ used for point extraction. The non-rigid differences (errors) calculated as the difference due to misalignment between reference and moving images are reported for each slice in Fig. 6a. ‘Registration 2’ gives the best approximation, showing less non-rigid errors compared with ‘Registration P’. Therefore, we use ‘Registration 2’ for calculating the non-rigid displacement and strain fields, by subtracting the displacements obtained with ‘Registration 1’ from the ones calculated with ‘Registration 2’.

Figure 6b shows the deformations in pixel, where a pixel measures 0.8 μm, in the tangential (*x*-) and radial (*y*-) directions for one slice of the datasets. The deformations are calculated over the whole area of the ROI, and thus deformations are calculated independently from the actual cellular structure. In the next step, shown in Fig. 6c, the total local strain (affine plus non-rigid), are presented. In this sample, a combination of positive (red) and negative (blue) total strains in *E*_*xx*_ are observed in the region between earlywood and latewood. This combined effect indicates a bending of the cell structure. For *E*_*yy*_, a negative strain (blue) is observed along the location of ray cells in the earlywood cell wall, surrounded by regions in earlywood with positive (red) strains. This observation could indicate to a kind of slip behaviour between rays and surrounding material. This result suggests the restraining role of ray cells on the cellular structure of soft materials, such as wood.

**Fig. 7:**
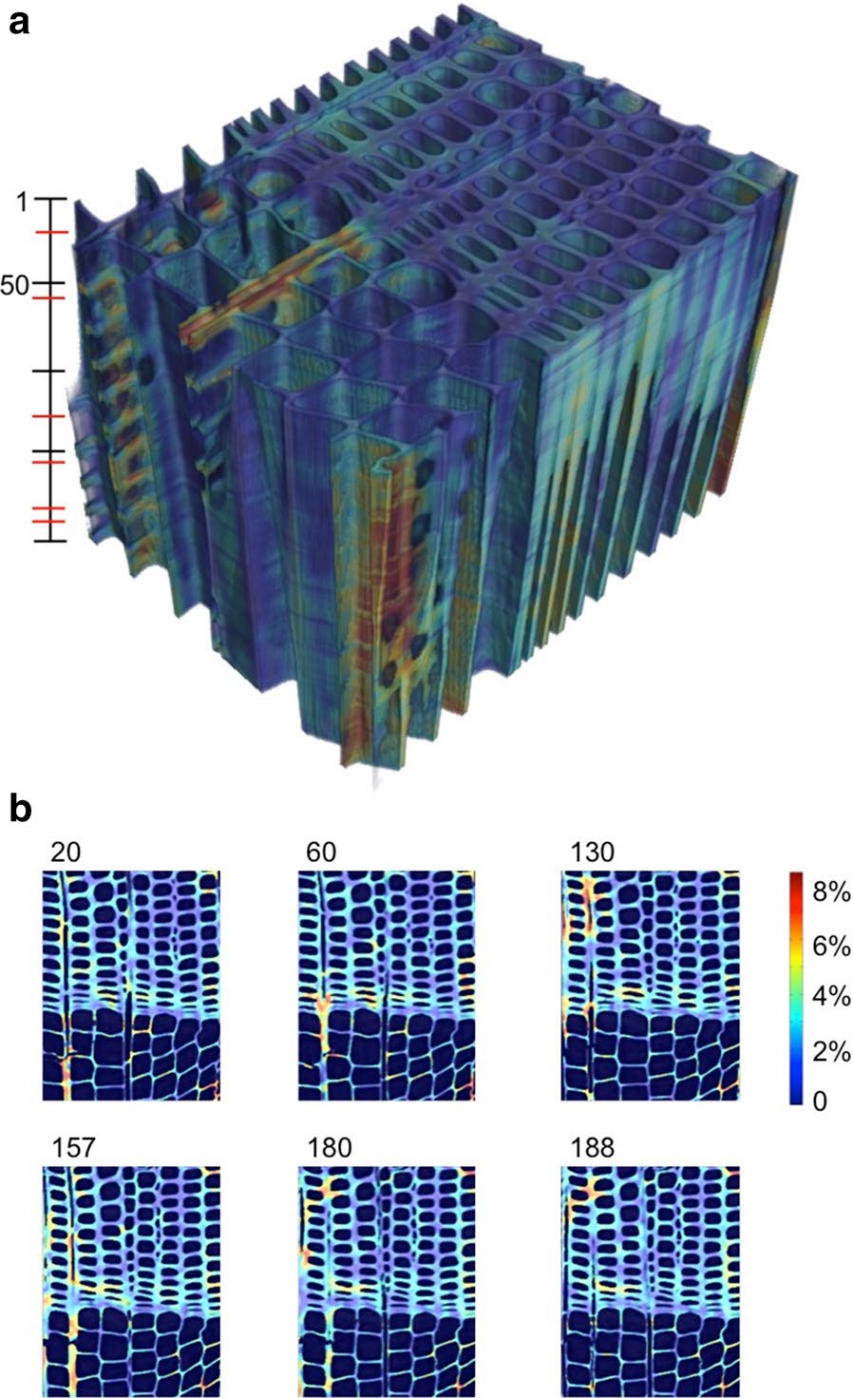
**a** Three-dimensional map of equivalent von Mises non-rigid strains on the wood cell wall of sample ELW_*d*,1_ for 200 slices, as indicated in the bar. **b** Cross-section cuts in the position indicated in red in the bar

Finally, the equivalent non-rigid (von Mises) components are calculated slice by slice and plotted in 3D respecting the cellular structure in Fig. 7. Figure 7 shows that high values of equivalent non-rigid strains occur in latewood cells close to the latewood/earlywood interface and close to the ray cells, especially in the earlywood layer. Wood samples combined by high density (named latewood) and low density (earlywood) swell in general less than the single tissues. In addition, it was found in Patera et al. [25] that the swelling becomes less anisotropic between radial and tangential directions in combined samples compared to single earlywood and latewood tissues. This means that, due to the restraining effect of latewood on earlywood and vice versa, swelling of combined wood reduces and becomes less anisotropic. In the analysis of the non-rigid strain components, it is possible to observe positive swelling strain values in latewood cells located at the interface latewood/earlywood and shrinkage strains in soft earlywood cells. This effect is especially visible in the tangential direction of wood. This means that, in these regions of combined compressive/tensile stresses, a local bending with respect to the interface late-/earlywood occurs. Such local bending effects or high strain effects around restraining rays cannot be observed by global affine analysis. Further applications of this proposed non-rigid registration method are presented in Patera et al. [26].

The results presented in this section illustrate clearly that non-rigid registration is a powerful tool for capturing the deformations of complex cellular and biological materials. Such information cannot be recovered using other types of image registration techniques, such as, for example, the affine registration.

## Conclusion

In this work, a new algorithm is developed based on the work of Rueckert using B-spline. The main contribution consists in an accurate combination for the specific case study between the concept of feature recognition within specific regions locally distributed in the material with an optimization problem. The work is validated with a synthetically deformed dataset in bending and is used for documenting local swelling on a complex cellular material, wood, using two states of moisture content at 25 and 85% RH.

The non-rigid registration algorithm introduced in this work is a powerful tool for detecting and quantifying the non-rigid deformations in complex biological materials, such as wood. The algorithm contains a wide range of tools for image analysis: in particular, morphological operations, segmentation and linear and non-linear transformations. This work is mainly focused on the implementation of a non-linear transformation based on B-spline functions. The method can be used for studying the behaviour of different existing materials detected with several experimental setups, in 2D, and it can be extended to a 3D case study.

In this work, the technique is used to investigate the occurrence of local deformations in wood provoked by moisture changes. The datasets are acquired with X-ray Tomography, thus leading to little changes in the grey-value intensity within the cell walls. This allows the use of squared sum of intensity differences (SSD) as similarity criterion. However, there are cases, especially in medicine, where the two datasets, i.e., reference and moving, are acquired with different configurations leading to contrast enhancement (i.e., pre- and post-contrast agent with magnetic resonance imaging). In such case, another similarity criterion, insensitive to intensity changes, would be more suitable, as already demonstrated in Rueckert et al. [28]. The proposed method is expected to be widely applicable in material science and in medicine for detecting locally object deformations.

## References

[1] Abd-Elmoniem KZ, Stuber M, Prince JL (2008). Direct three- dimensional myocardial strain tensor quantification and tracking using zHARP. Med. Image Anal..

[2] Brown LG (1992). A survey of image registration techniques. ACM Comput. Surv..

[3] Canny J (1986). A computational approach to edge detection. IEEE Trans. Pattern Anal. Mach. Intell. PAMI.

[4] Collins DL, Evans AC (1997). Animal: validation and applications of nonlinear registration-based segmentation. Int. J. Pattern Recognit Artif Intell..

[5] Derome D, Griffa M, Koebel M, Carmeliet J (2011). Hysteretic swelling of wood at cellular scale probed by phase-contrast X-ray tomography. J. Struct. Biol..

[6] Dierick M, Van Loo D, Masschaele B, Van den Bulcke J, Van Acker J, Cnudde V, Van Hoorebeke L (2010). Recent micro-CT scanner developments at UGCT. Nucl. Instrum. Methods Phys. Res. Sect. B.

[7] Dornheim, L., Tönnies, K. D., Dixon, K.: Automatic segmentation of the left ventricle in 3D SPECT data by registration with a dynamic anatomic model. In: Duncan, J. S., Gerig, G. (eds.) Medical image computing and computer-assisted intervention—MICCAI 2005, pp. 335–342. Springer, Berlin (2005). http://link.springer.com/chapter/10.1007/11566465_42 10.1007/11566465_42 16685863

[8] Foskey M, Davis B, Goyal L, Chang S, Chaney E, Strehl N, Tomei S, Rosenman J, Joshi S (2005). Large deformation 3D image registration in image-guided radiation therapy. Phys. Med. Biol..

[9] Frangi AF, Laclaustra M, Lamata P (2003). A registration-based approach to quantify flow-sequences. IEEE Trans. Med. Imaging.

[10] Gao Y, Sandhu R, Fichtinger G, Tannenbaum AR (2010). A coupled global registration and segmentation framework with application to magnetic resonance prostate imagery. IEEE Trans. Med. Imaging.

[11] Gering, D. T., Nabavi, A., Kikinis, R., Grimson, W. E. L., Hata, N., Everett, P., Wells, W. M.: An integrated visualization system for surgical planning and guidance using image fusion and interventional imaging. In: Taylor, C., Colchester, A. (eds.), Medical image computing and computer-assisted intervention—MICCAI’99, pp. 809–819. Springer, Berlin (1999). http://link.springer.com/chapter/10.1007/10704282_88

[12] Gering DT, Nabavi A, Kikinis R, Hata N, O’Donnell LJ, Grimson WEL, Wells WM (2001). An integrated visualization system for surgical planning and guidance using image fusion and an open MR. J. Magn. Reson. Imaging.

[13] Gooya A, Biros G, Davatzikos C (2011). deformable registration of glioma images using EM algorithm and diffusion reaction modeling. IEEE Trans. Med. Imaging.

[14] Harris, C., Stephens, M.: A combined corner and edge detector. In: Alvey vision conference, 15.50. Manchester, UK (1988). http://courses.daiict.ac.in/pluginfile.php/13002/mod_resource/content/0/References/harris1988.pdf

[15] Huang X, Ren J, Guiraudon G, Boughner D, Peters TM (2009). Rapid dynamic image registration of the beating heart for diagnosis and surgical navigation. IEEE Trans. Med. Imaging.

[16] Isgum I, Staring M, Rutten A, Prokop M, Viergever MA, Bv Ginneken (2009). Multi-atlas-based segmentation with local decision fusion—application to cardiac and aortic segmenta- tion in CT scans. IEEE Trans. Med. Imaging.

[17] Lavely WC, Scarfone C, Cevikalp H, Li R, Byrne DW, Cmelak AJ, Fitzpatrick JM (2004). Phantom validation of coregistration of PET and CT for image-guided radiotherapy. Med. Phys..

[18] Lee S, Wolberg G, Shin SY (1997). Scattered data interpolation with multilevel B- splines. IEEE Trans. Visual Comput. Graphics.

[19] Maksimov D, Hesser J, Brockmann C, Jochum S, Dietz T, Schnitzer A, Diehl S (2009). Graph-matching based CTA. IEEE Trans. Med. Imaging.

[20] Martin S, Daanen V, Troccaz J (2008). Atlas-based prostate segmentation using an hybrid registration. Int. J. Comput. Assist. Radiol. Surg..

[21] Masschaele BC, Cnudde V, Dierick M, Jacobs P, Van Hoorebeke L, Vlassenbroeck J (2007). UGCT: new X-ray radiography and tomography facility. Nucl. Instrum. Methods Phys. Res. Sect. A.

[22] Miller K, Wittek A, Joldes G, Horton A, Dutta-Roy T, Berger J, Morriss L (2010). Modelling brain deformations for computer-integrated neurosurgery. Int. J. Numer. Methods Biomed. Eng..

[23] Mises, R.v.: Mechanik der festen Körper im plastisch-deformablen Zustand. Nachrichten von des Gesellschaft des Wissenschaften zu Göttingen, Mathematisch-Physicalisch Klasse **1913**, 582–592 (1913)

[24] Nocedal J, Wright S (2006). Numerical Optimization.

[25] Patera A, Derome D, Griffa M, Carmeliet J (2013). Hysteresis in swelling and in sorption of wood tissue. J. Struct. Biol..

[26] Patera A, Van den Bulcke J, Boone M, Derome D, Carmeliet J (2017). Swelling interactions of earlywood and latewood across a growth ring: global and local deformations. Wood Sci. Technol..

[27] Pluim JPW, Maintz JBA, Viergever MA (2003). Mutual-information-based registration of medical images: a survey. IEEE Trans. Med. Imaging.

[28] Rueckert D, Sonoda LI, Hayes C, Hill DLG, Leach MO, Hawkes DJ (1999). Nonrigid registration using free-form deformations: application to breast MR images. IEEE Trans. Med. Imaging.

[29] Sederberg T, Parry S (1986). Free-form deformation of solid geometric models. Proc. ACM SIGGRAPH.

[30] Staring M, van der Heide UA, Klein S, Viergever MA, Pluim J (2009). Registration of cervical MRI using multifeature mutual information. IEEE Trans. Med. Imaging.

[31] Szeliski R, Coughlan J (1997). Spline-based image registration. Int. J. Comput. Vision.

[32] Wahba, G.: Spline models for observational data. Soc. Ind. Appl. Math (1990). http://epubs.siam.org/doi/book/10.1137/1.9781611970128

[33] Wyawahare MV, Patil PM, Abhyankar HK (2009). Image registration techniques: an overview. Int. J. Signal Process. Image Process. Pattern Recogn..

[34] Zhuang X, Rhode KS, Razavi RS, Hawkes DJ, Ourselin S (2010). A registration-based propagation framework for automatic whole heart segmentation of cardiac MRI. IEEE Trans. Med. Imaging.

